# Growing old in China in socioeconomic and epidemiological context: systematic review of social care policy for older people

**DOI:** 10.1186/s12889-023-15583-1

**Published:** 2023-06-30

**Authors:** Sophia Lobanov-Rostovsky, Qianyu He, Yuntao Chen, Yuyang Liu, Yanjuan Wu, Yixuan Liu, Tishya Venkatraman, Eric French, Natasha Curry, Nina Hemmings, Piotr Bandosz, Wing Kit Chan, Jing Liao, Eric John Brunner

**Affiliations:** 1 Department of Epidemiology & Public Health, University College London, London, WC1E 7HB UK; 2 Department of Medical Statistics & Epidemiology, School of Public Health, Sun Yat-sen University, Guangzhou, 510275 P.R. China; 3 Sun Yat-sen Global Health Institute, School of Public Health, Institute of State Governance, Sun Yat-sen University, Guangzhou, 510275 P.R. China; 4 Shenzhen Health Development Research and Data Management Center, Shenzhen, China; 5 Faculty of Economics, University of Cambridge, CB3 9DD Cambridge, UK; 6 Institute for Fiscal Studies, University of Cambridge, London, WC1E 7AE UK; 7 Policy Department, Nuffield Trust, W1G 7LP London, UK; 8 Department of Prevention and Medical Education, Medical University of Gdansk, Gdansk, 80-210 Poland; 9 School of Government, Sun Yat-sen University, Guangzhou, 510275 P.R. China

**Keywords:** Ageing, China, Systematic review, Health equity, Social care

## Abstract

**Background:**

From 2020 to 2050, China’s population aged ≥65 years old is estimated to more than double from 172 million (12·0%) to 366 million (26·0%). Some 10 million have Alzheimer’s disease and related dementias, to approach 40 million by 2050. Critically, the population is ageing fast while China is still a middle-income country.

**Methods:**

Using official and population-level statistics, we summarise China’s demographic and epidemiological trends relevant to ageing and health from 1970 to present, before examining key determinants of China’s improving population health in a socioecological framework. We then explore how China is responding to the care needs of its older population by carrying out a systematic review to answer the question: ‘what are the key policy challenges to China achieving an equitable nationwide long-term care system for older people?’. Databases were screened for records published between 1st June 2020 and 1st June 2022 in Mandarin Chinese or English, reflecting our focus on evidence published since introduction of China’s second long-term care insurance pilot phase in 2020.

**Results:**

Rapid economic development and improved access to education has led to widescale internal migration. Changing fertility policies and household structures also pose considerable challenges to the traditional family care model. To deal with increasing need, China has piloted 49 alternative long-term care insurance systems. Our findings from 42 studies (n = 16 in Mandarin) highlight significant challenges in the provision of quality and quantity of care which suits the preference of users, varying eligibility for long-term care insurance and an inequitable distribution of cost burden. Key recommendations include increasing salaries to attract and retain staff, introduction of mandatory financial contributions from employees and a unified standard of disability with regular assessment. Strengthening support for family caregivers and improving smart old age care capacity can also support preferences to age at home.

**Conclusions:**

China has yet to establish a sustainable funding mechanism, standardised eligibility criteria and a high-quality service delivery system. Its long-term care insurance pilot studies provide useful lessons for other middle-income countries facing similar challenges in terms of meeting the long-term care needs of their rapidly growing older populations.

**Supplementary Information:**

The online version contains supplementary material available at 10.1186/s12889-023-15583-1.

## Background

China’s population of 1·44 billion is ageing fast. People aged ≥65 years old account for 12·0% (173 million) of the population, and this proportion is expected to rise to some 26·1% (366 million) by 2050 (Table 1) [1]. Population ageing is firmly on the national policy agenda but continues to be a major challenge, with both generic and particularly Chinese characteristics. In common with many countries, the rapidly ageing population is a source of fiscal stress and workforce shortfall. More specifically, inequalities in socioeconomic development across China’s vast population and land area make it difficult to support the care needs and wellbeing of all its older people.

**Fig. 1:**
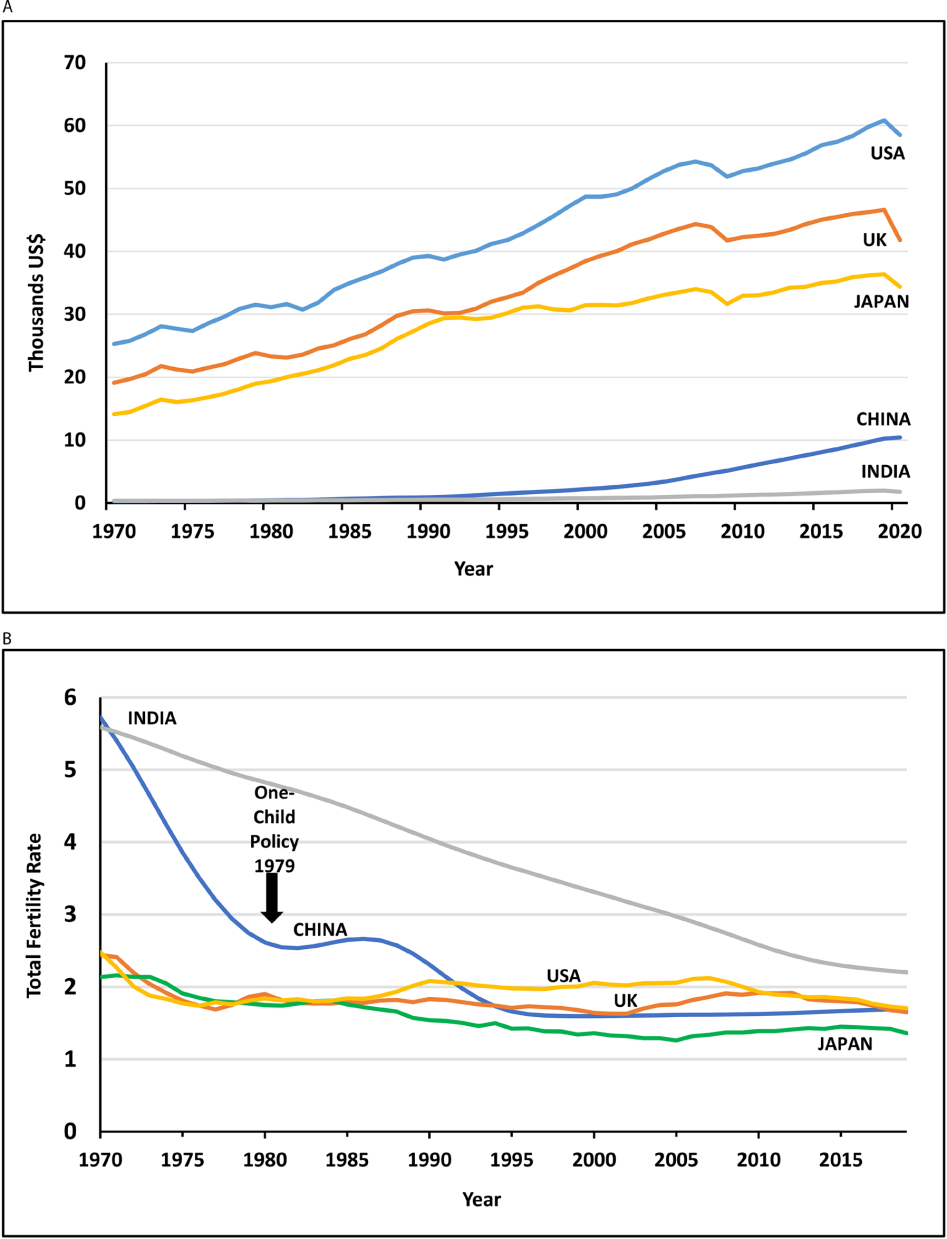
Trends in standard of living and total fertility rate, China, India, UK, USA and Japan, 1970–2020 A: Gross Domestic Product per capita (constant 2015 US$) of China, India, UK, USA and Japan, 1970–2020 *Source: World Development Indicators. The World Bank* [5]. Footnote: GDP per capita is Gross Domestic Product divided by by mid-year population. Data are in constant 2015 US$. B: Total fertility rate for China, India, UK, USA and Japan, 1970–2019 *Source: World Development Indicators. The World Bank* [8]. Footnote: Total fertility rate in a specific year is the total number of children that would be born to each woman if she were to live to the end of her child-bearing years and give birth to children according to prevailing age-specific fertility rates.

Critically for China, its population is ageing while it is still a middle-income country. This dynamic means China faces ‘being old before being rich’ [2, 3]. Gross Domestic Product (GDP) per capita increased from 405 US$ in 1979 to 10,431 US$ in 2020 (Fig. 1A), with a long-run economic boom that peaked in 2007 (13·6% GDP growth per employed person) [4]. China is expected to reach the high income threshold (US$12,700 per capita) in 2025, but will still be years away from expenditure and consumption levels of G7 countries.

Among the G7, Japan introduced universal long-term care insurance (LTCI) in 2000. In contrast to China, Japan ‘grew rich before growing old’. Japan nevertheless struggles to fund the social care it aspires to provide [5, 6]. Japan’s experience highlights the challenge for China to develop a financially sustainable social care system in the context of lower per capita income.

Comparing China to India, both middle-income countries with extensive geography and population, China’s economic growth and population ageing is considerably faster (Table 1). China’s decline in fertility rate has outdone India’s for five decades, approximately equals that of high income countries such as the United Kingdom (UK) and the United States of America (USA), and approached that of Japan in 1995 (Fig. 1B) [7]. Cohorts born in China’s 1950s and 1960s baby boom are reaching retirement age, with replacement by smaller cohorts born in the 1990s [3]. China’s shrinking workforce is occurring at the same time as the need to drive up age-related social care capacity.

Comparison of China with the USA, UK, Japan and India, demonstrates a need to understand the social policy implications of population ageing in both generic and unique aspects of the Chinese context.

We aim to achieve this by summarising China’s key demographic and epidemiological trends relevant to ageing and health from 1970 to present, using official and population-level statistics, and demonstrate how major socioeconomic reforms since 1970 have contributed to China’s improving population health in a socioecological framework [8]. We explore their shaping of social care policy for the older population, and carry out a systematic review which aims to answer the question: ‘what are the key policy challenges to China achieving an equitable nationwide long term care (LTC) system for older people?’.

## China’s population health trends

### Demography

Over the past 50 years, China’s population has grown by >600 million with dramatic shifts in age structure. In 1970, 51·7% of the Chinese population was <20 years old and only 3·7% was >65 years old (Table 1). China and India had a similar old-age dependency ratio (OADR, 0·08, 0·07, respectively), and >50.0% of each country’s population were children or adolescents (0–19 years). By 2020, the proportion of the population <20 years old had fallen to 23·4%, the same level as the UK. China’s OADR was almost twice that in India. Projections estimate China’s >65 year old population to almost double that of India by 2050 and exceed that of the UK and the USA. By 2050, China’s OADR is expected to reach that of the UK, and to approach that of Japan in 2020 (respectively, 0·48 and 0·52).

The working age population is predicted to decline by 23% from 925 million in 2011 to 800 million in 2050 [9]. Exacerbating the problem of population ageing, retirement ages in China are lower than in many other countries.

At the same time, China’s total fertility rate (TFR) of 5·6 in 1970 declined rapidly to levels below the population replacement rate of 2·1 by 1991. Contraction of the size of younger birth cohorts after 1991, individuals currently ≤30 years old, is a key part of China’s demographic shift [2].

China’s TFR currently stands at an estimated value of 1·3 (12 million births per year), according to the Seventh National Population Census, published in 2021 [10]. The World Bank estimates a higher value of 1·7 (Fig. 1B) [7]. The discrepancy is largely attributed to differences in model estimation, where counting births accurately across all Chinese territory was subject to error [11]. For verification, the Seventh Census used administrative records e.g. citizenship information and big data sources, with a low missing rate (0·05%) [12]. China’s TFR is expected to remain stable for the next three decades [7]. However, skewed sex ratios at birth make TFR prediction difficult [13]. In 2020, the United Nations (UN) reported 113 males per 100 females, with values expected to remain skewed into 2050 [14].

**Table 1:** Population, age structure and old-age dependency ratio in 1970 and 2020, China, India, Japan, UK, and USA

	Population, million	0–19 years, million (%)	20–64 years, million (%)	≥65 years, million (%)	Old-age dependency ratio	
**China**						
1970 2020	827·6 1,439·3	427·6 (51·7) 337·2 (23·4)	369·0 (44·6) 929·8 (64·6)	31·0 (3·7) 172·3 (12·0)	0·08 0·19	
2050	1,402·4	266·5 (19·0)	769·9 (54·9)	366·0 (26·1)	0·48	
**India**						
1970	555·2	285·7 (51·5)	251·2 (45·2)	18·3 (3·3)	0·07	
2020	1,380·0	487·1 (35·3)	802·2 (58·1)	90·7 (6·6)	0·11	
2050	1,639·2	413·1 (25·2)	1001·5 (61·1)	226·2 (13·8)	0·23	
**Japan**						
1970	104·9	34·6 (33·0)	63·1 (60·2)	7·2 (6·9)	0·11	
2020	126·5	21·5 (17·0)	69·1 (54·6)	35·9 (28·4)	0·52	
2050	105·8	16·5 (15·6)	49·4 (46·7)	39·9 (37·7)	0·81	
**UK**						
1970	55·6	17·3 (31·1)	31·1 (55·9)	7·2 (12·9)	0·23	
2020	67·9	15·6 (23·0)	39·6 (58·3)	12·7 (18·7)	0·32	
2050	74·1	15·4 (20·8)	39·9 (53·8)	18·7 (25·3)	0·47	
**USA**						
1970	209·5	78·3 (37·4)	110·1 (52·6)	21·1 (10·1)	0·19	
2020	331·0	82·1 (24·8)	193·9 (58·6)	55·0 (16·6)	0·28	
2050	379·4	84·6 (22·3)	210·2 (55·4)	85·0 (22·4)	0·40	

Source: Data from United Nations, Department of Economic and Social Affairs, Population Division (2019). World Population Prospects 2019.

Footnote: Limited demographic data is available for China before 1970. Old age dependency ratio is the proportion of people aged ≥65 (generally economically inactive) relative to those aged 15–64 years old. Normal retirement age (NRA) varies between countries, blurring the boundary between economically active and inactive individuals. The NRA for both men and women is 66 in the UK and the USA, 65 in Japan, 58 in India. In China, the NRA is 60 for men and 55 for women [15].

### All-cause mortality, life expectancy and China’s epidemiological transition

China’s population health trends follow the classical demographic transition linked with a country’s economic development. Since 1990, non-communicable diseases (NCDs) have largely replaced communicable diseases, with five risk factors (high fasting plasma glucose, high blood pressure, high body-mass index, smoking and air pollution) becoming dominant [16].

Improved maternal education, greater access to antenatal care and immunisation have resulted in a substantial reduction in infant and child mortality rates since 1990 (average annual declines of 6·2% and 6·6%, respectively). China achieved Millennium Development Goal 4, a two thirds reduction in under-5-mortality rate, by 2008, seven years before its target date [17].

Adult mortality (20–64 years) has declined on average 2·3% per year between 1990 and 2019 (Table 2). For older people (≥65 years old) the average annual decline was 1·6%. Life expectancy at age 65 increased from 13·8 to 17·2 years between 1990 and 2019, with increasing female life expectancy advantage [18].

A global mortality shock, induced by the SARS-CoV-2 (COVID-19) pandemic, has impacted health trends. Since December 2019, the pandemic has resulted in some 15 million excess deaths globally [19]. Inconsistent death registration complicates estimates of COVID-19 deaths. Excess deaths (the difference between observed and expected deaths in a time period) is the most reliable, though not cause-specific, way to manage this issue [20].

On 6th December 2022, China’s official statistics reported 5,235 COVID-related deaths. Lifting of the zero-COVID policy on 7th December led to an estimated 60,000 excess deaths in the following month [21]. The Global Burden of Disease (GBD) Study estimates that excess deaths will reach 322,000 by April 2023 [22].

Older people, and those with comorbidities, are particularly vulnerable to severe infection. In China, early stages of the pandemic (April-May 2020), led to a case-fatality ratio of 18·5% among 70–79-year-olds, increasing to 32·1% in those ≥80 years old [23]. Average age of death is 80 years old; 90·1% of fatalities have occurred in those aged ≥65. This follows the age-related pattern of excess deaths observed for other respiratory illnesses such as influenza [24].

The impact of excess deaths on life expectancy is still unknown. In the Spanish flu pandemic (1918-20), life expectancy declined continuously among all countries with available data but recovered within one-to-two years. Life expectancy losses in 2020 bounced back to pre-COVID-19 levels within a year in some European countries (Sweden, Switzerland and Belgium). Recovery is attributed to public health measures aimed at reducing mortality among those aged ≥60, notably COVID-19 vaccination [25].

As of November 2022, some 65·8% of Chinese adults ≥80 years old had received a COVID-19 vaccination, and only 40% had received a booster [26]. Vaccine hesitancy is associated with concerns for safety, effectiveness, and lack of knowledge: Chinese adults aged ≥60 are more likely to trust media, relatives and friends for COVID-19 vaccination information [27]. It is plausible that low vaccination rates will stall China’s life expectancy improvements.

Low vaccination uptake also increases older people’s risk to long-lasting, adverse effects of infection. One study in Shandong province (n = 255) showed that more than half of unvaccinated patients experienced post-COVID-19 symptoms and abnormal chest scans one year after hospital discharge [28]. Cognitive impairment was found in 12·5% of COVID-19 survivors ≥60 years old (n = 3233), with no pre-existing conditions, 12 months after infection [29].

Travel restrictions have reduced viral transmission, however serious public health concerns arise over heightened risks of social isolation and loneliness among older adult populations [30]. High psychological distress (a score of ≥22 on the Kessler Psychological Distress Scale) increased by 7·8% among rural older adults aged ≥60 in Shandong province (n = 2749) between May 2019 and August 2020 [31]. This risk was higher among those who became isolated during the pandemic.

**Table 2:** Age-standardised all-cause mortality rates and life expectancy in China, 1990–2019

	1990	2019	Mean annual change
**All-cause mortality rate, per 100 000**			
Infant (<1 year)	4318	676·5	-6·2%
Child (<5 years)	1154	160·4	-6·6%
Adult (20–64 years)	564·4	285·6	-2·3%
Older (≥65 years)	6888	4354	-1·6%
**Life expectancy at birth, years**			
Total	68·1	77·6	0·33
Men	66·2	74·7	0·30
Women	70·2	80·8	0·36
**Life expectancy at 65, years**			
Total	13·8	17·2	0·12
Men	12·7	15·4	0·09
Women	14·8	19·1	0·15

Source: Global Burden of Disease Results Tool http://ghdx.healthdata.org/gbd-results-tool

Footnote: Age-standardized all-cause mortality rates for adults based on age distribution of 2019 Chinese population

### Age-related disability

A three-fold increase in ≥65 year old population between 1970 and 2020 (Table 1) has driven a rapid rise in age-related disability. Estimates of the number (%) of older adults (aged ≥60) living with disability range between 33 and 45 million (19–26%) [32, 33].

Functional impairment and disability at older ages may be caused by one or more chronic diseases, including Alzheimer’s disease and related dementias (ADRD), which accounted for about 10% (ADRD estimate: 10·4 million) in China in 2016 [34]. Prevalence of ADRD is highest among those ≥80 years old (12%) [35]. A meta-analysis of Chinese surveys, taking account of methodological and geographical differences, produced a similar estimate (9·5 million) [36]. A model-based prediction put the estimate of ADRD at 16 million in 2020, increasing to 49 million in 2050 [37]. Even if ADRD incidence rates declined by 10% per decade over the coming three decades, the number of cases would approach 40 million by 2050 [37].

Cardiovascular disease (CVD), and particularly stroke, is the leading cause of disability-adjusted life years (DALYs) in China. In 2018, 290 million were living with CVD (an increase of 60 million from 2007) [38]. According to the GBD Study, prevalence of CVD among ≥80 year olds increased by 5·5% from 1990 to 2019 (47·3% to 52·8%). 28·8 million people are currently living with stroke (ischaemic stroke, 24·1 million) [39].

Hypertension and diabetes are major risk factors for vascular disease and disability. The China Hypertension Survey (2012-15) found a high prevalence of hypertension in ≥75 year olds (60%) [40]. Prevalence of diabetes is 12.4% among Chinese adults aged ≥18, compared with a global estimate of 8·3%, and ranked highest in the world (89·5 million) [39–42]. A national survey estimated prevalence of diabetes in 16.5% of adults aged ≥70 [42].

Based on these disease trends, and the predicted size of the older population, the number with disability (aged ≥60) is set to increase further by 2050 [43].

### Health inequalities

The burden of age-related disability is heterogenous across China. Rural China has a higher age- and sex-standardised prevalence of ADRD than urban regions in the age ≥65 population (rural 6·1% vs. urban 4·4%) [44, 45]. Cities attract younger people with higher education levels, for higher wages and standard of living. These indicators are associated with good health, and lower risk of chronic disease [46]. Lower income and education level, both associated with rural residence, are barriers to health care access [47]. Consistent with this pattern, the Chinese Longitudinal Healthy Longevity Survey (CLHLS) observed a higher prevalence of good or very good self-rated health among urban residents aged ≥80 than their rural counterparts in 2018 (50·2% vs. 45·1%) [48].

Disparities between rural and urban regions are more broadly influenced by China’s geographic diversity and differing levels of socioeconomic development. China has high geographic inequality in health care resources: institutions, health workers and beds [49]. The more developed, southern and eastern provinces generally have better health compared to the mountainous western and northern regions, where resources are sparse [50].

The mortality rate in the eastern province of Shanghai was half that in the western province of Qinghai in 2018 (391 vs. 796 per 100,000) [51] Age-standardised years of life lost per 100,000 were dramatically higher in Qinghai than Shanghai (2700 vs. 650 per 100,000) [51]. In 2012, under-5 mortality in Shanghai was lower than the USA and New Zealand. Comparatively, rates were 32 times greater in Sichuan province (western China), exceeding that of Bangladesh, and similar to Burkina Faso [52].

Estimated prevalence of dementia in the ≥65 year old population is far higher in western China (7·2% vs. 4·8% in southern China) [53]. 38·6% of adults ≥60 years old in the western regions live with depression, compared to 26·6% in the East [54]. Estimated stroke prevalence is nearly 2.5 times higher in northeastern compared to south-eastern areas. Prevalence of CVD is 12·6% in northeast China and 8·0% in the South [55, 56].

Health inequalities by region are further determined by the country’s ethnic and cultural diversity. The 2020 national census estimated that Han Chinese accounted for 91·1% (1286 million) of the population, with the remaining 55 ethnic groups accounting for 8·9% (125 million), the largest being Zhuang, Hui, Manchu and Uygur [57]. Less developed regions are more ethnically diverse. Of China’s 56 ethnic groups, 44 are concentrated in China’s western provinces [57]. Despite improvements, ethnic minority health status continues to lag behind that of the majority Han Chinese population. Self-rated health is poorer among ethnic minority groups [58]. Low socioeconomic status (poverty, low income, low-status employment) and education level are associated with poorer health outcomes [58].

Although women live longer (Table 2), they tend to report more symptoms and receive more diagnoses than men. In 2015, an estimated prevalence of multimorbidity in older women (aged ≥60) was 57% compared to 46% in older men [59]. As women tend to outlive their husbands, they are more likely to receive poor quality care and have unmet care needs [60].

### Determinants of China’s population health

**Fig. 2:**
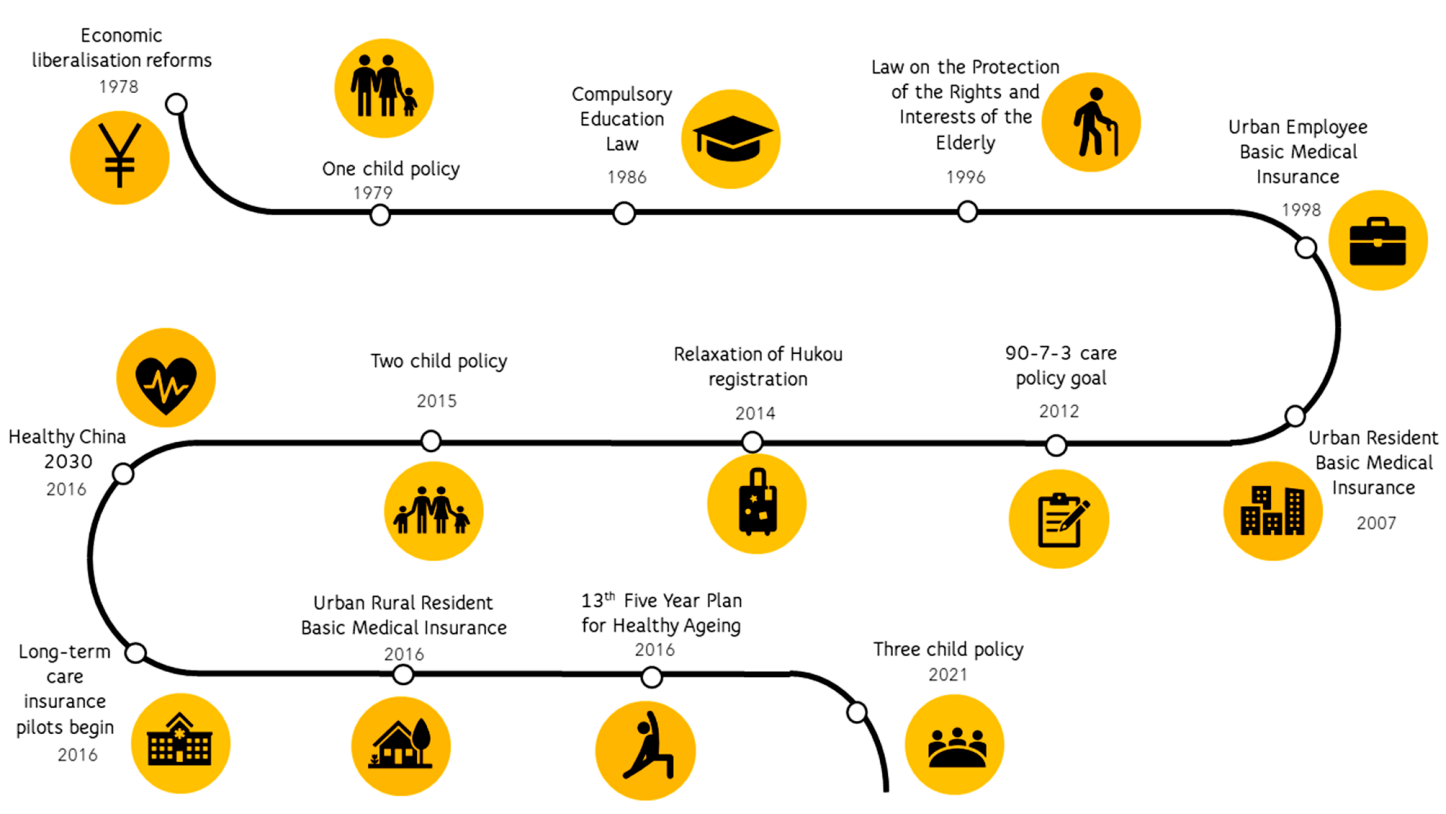
Population health milestones in China, 1978–2021

**Fig. 3:**
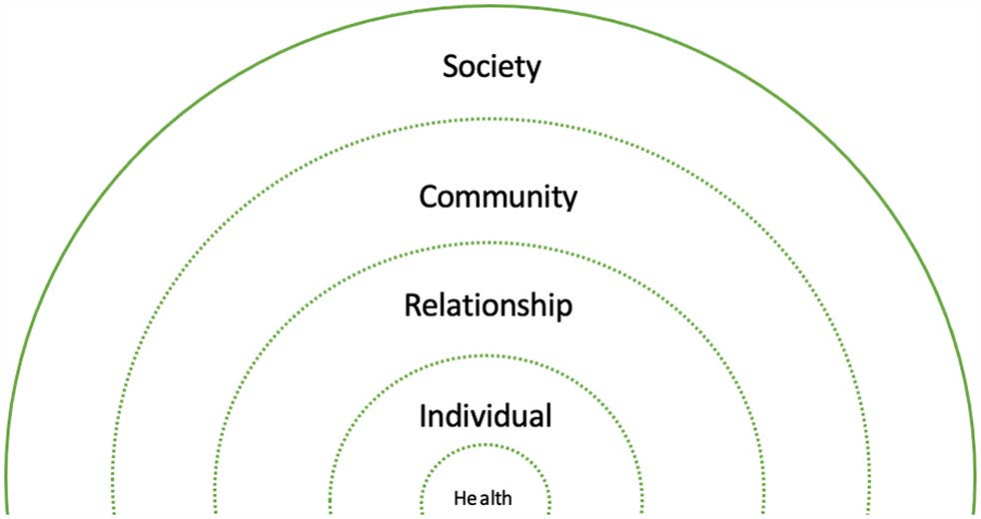
Socioecological framework of population health *Source: Adapted from Dahlgren and Whitehead, Lancet, 1991.* [9] Footnote: Dotted lines between the levels represent the complex interaction of determinants. Societal factors concern social, economic, cultural and environmental conditions. Community considers the characteristics of neighbourhoods, regions, schools and the workplace while relationship refers to person-to-person interactions such as social networks and family. Individual factors concern personal characteristics, genetics, lifestyle and behavioural factors.

Social, educational, and economic reforms over the last 50 years have led to dramatic improvements in China’s population health (Fig. 2). Using a socioecological framework (Fig. 3), we examine how key reforms have impacted social determinants of health, and shaped social care policy for the older population [61].

## Society

### Economic reforms

Wealth and income are each closely associated with improvements in health [62]. Older Chinese people who report excellent or very good health have on average twice the household income of those reporting worse health. A strong inverse association between household income and poor self-rated health was observed in the 2006 Chinese Health and Nutrition Survey, representative of nine provinces and 42% of the population [63].

Economic growth in China accelerated after introduction of reforms in 1978 [2]. The largely isolated economy grew by some 4·4% per year in purchasing power parity between 1952 and 1979. Introduction of the ‘Open Door Policy’ in 1978 opened China to foreign investment. ‘Special export zones’ for foreign trade were established in Guangdong province and Fujian province. China joined the World Trade Organisation in 2001 [64]. The rural economy was boosted through introduction of the Household Responsibility System in 1979, which decollectivized agriculture in favour of household-based rights to land use and to benefit from sale of agricultural products [65].

Since 1978, approximately 800 million people have been lifted out of poverty in China [66]. This decline is the result of higher average incomes, not a reduction in inequality. Income inequality, measured by the Gini coefficient, has been growing since the 1978 reforms and is now reported to be higher than in the USA [67]. In 2021, the urban-rural income ratio stood at 2·7 to 1 [68].

From 1990 to 2013, China contributed to falling rates of extreme poverty (1·90 US$ per day, per person), [70] making up nearly half of those in poverty globally, to 9·3% [69]. Rural poverty was reduced from 56 million to 17 million people between 2015 and 2018 [71]. The Chinese government allocated 91 billion RMB (13 billion US$, 11 billion GBP) to poverty alleviation funds for 2019 [69] and announced it had eliminated extreme rural poverty in February 2021 [66].

### Changing fertility policies

Expanding population size in the 1960s resulted in introduction of family planning policies and campaigns to alleviate severe poverty and promote economic growth [72]. The ‘Later, Longer, Fewer’ campaign, introduced in 1970, led to a dramatic reduction in TFR from 5·7 at its onset, to 2·7 in 1978 [73]. ‘Later’ referred to the encouragement of later marriage, ‘longer’ to greater intervals between births (at least four years) and ‘fewer’ meant a limit on the number of births permitted, at a maximum of two children for urban families and three for rural families [73].

Introduction of the one-child policy in 1979, coinciding with the 1978 economic reforms, preceded another downward move in TFR, after some delay (Fig. 1B). The TFR plateau may have resulted from variations in policy implementation and fertility rates across rural and urban areas, regions and ethnicities [72]. Although the one-child policy was the general principle, Han Chinese were expected to abide by the rules, while the fertility of ethnic minorities, most concentrated in western and less developed regions, was relatively unregulated [72]. The one-child policy was enforced and monitored in cities, government institutions and state-run businesses [72]. In contrast, the policy was largely unenforced in subsistence farming areas where there was strong resistance. Rules were relaxed in 1984, allowing rural couples to have a second child if their first born was a girl [74]. By 1991, TFR fell to the population replacement level of 2·1 [7].

Shrinkage of the working population led to the two-child policy in 2016, which meant some 90 million women became eligible to have a second child [75]. However, the number of births increased only in the first year, and has since declined. A three-child policy was introduced in May 2021 with mixed success: socioeconomic factors, including high costs of child-rearing, difficulty in accessing affordable childcare, along with women’s greater access to higher education and employment, have resulted in few women choosing to have large families [76].

### Compulsory education

During the lifetime of China’s present older population, the country has rapidly scaled the Preston Curve in terms of life expectancy (Table 2) [77]. Education is strongly associated with life expectancy, morbidity, health behaviours, labour market potential and health literacy [78].

Substantial improvements in education attainment resulted from introduction of nine years of compulsory education in 1986. From 1982 to 2010, the percentage of adults with a lower secondary education almost tripled (from 22·8% to 65·3%) [66]. By 2010, China’s secondary education enrolment reached 88·0%, closing the gap with other upper-middle-income countries such as South Africa and Brazil, despite lower GDP per capita [66].

Poor living conditions, an ageing teacher workforce, and lower education budgets in rural areas, mean education quality lags behind that of cities [79]. Households in the eastern regions have the highest average annual investment in primary and secondary education. Children in lower income households are more likely to drop out or repeat a school year [80]. China’s 14th Five Year Plan (2021-25) aims to narrow the urban-rural gap in access to quality education, including grouping schools under leadership of one outstanding school, and digitalisation of education [81].

## Community

### Internal migration

China’s export-orientated economy has generated huge demands for labour in urban regions. To meet this need, the central government relaxed the household registration system (*hukou*) in 2014, so that migrants could exchange their hukou status from that of origin to destination areas [82]. The hukou system was intended to reduce permanent rural-to-urban migration, protect agricultural production and promote societal equality in the face of rapid urbanisation [82]. It involved compulsory local registration at birth, as a rural or urban citizen, with resultant area-based eligibility for social services including health, education and welfare.

**Fig. 4:**
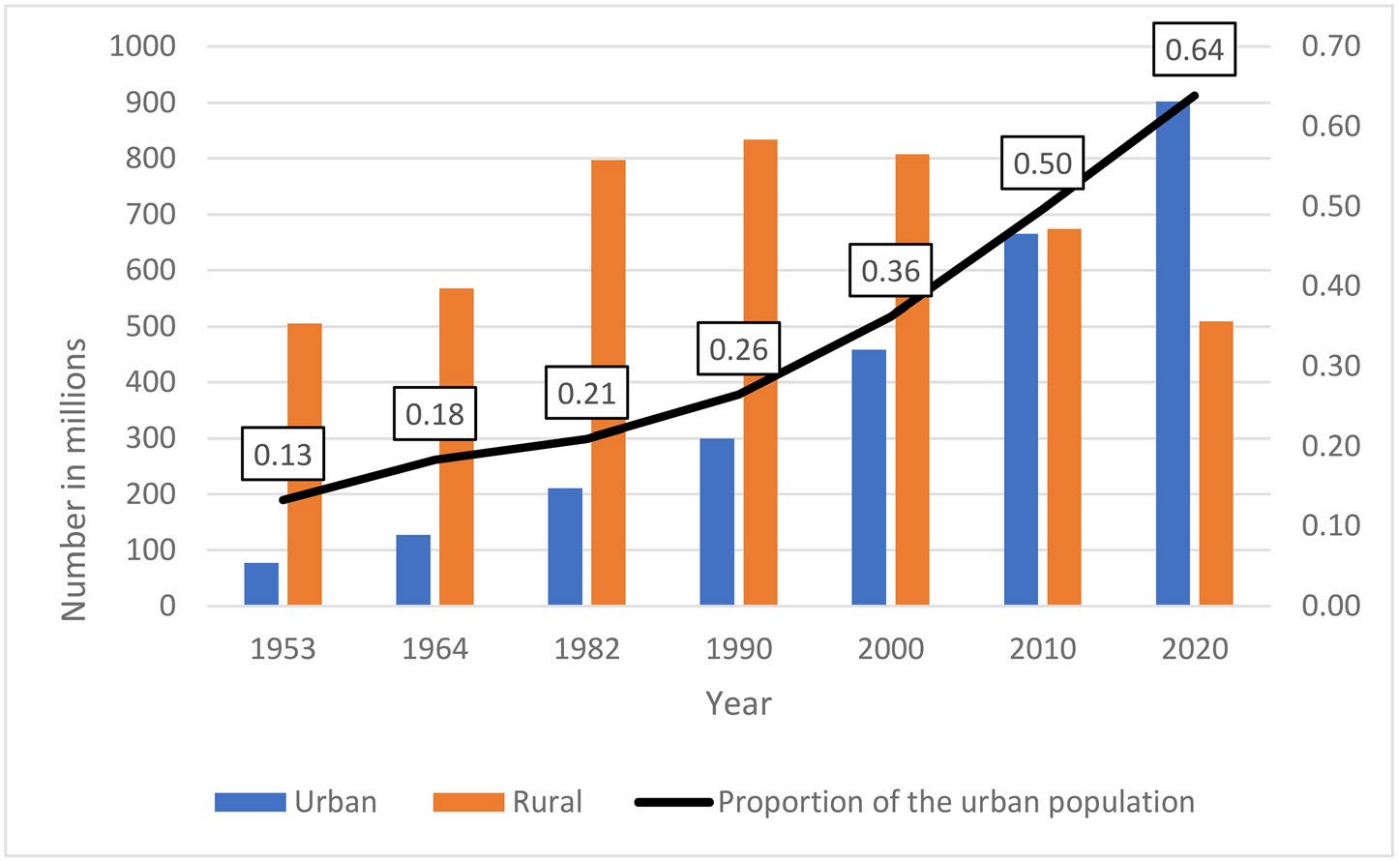
Chinese rural and urban populations, 1953–2020 *Source: National Bureau of Statistics. Bulletin of the Seventh National Population Census (No.7): Urban and Rural population and the Floating Population. 2021* [85].

Higher city wages and the changes to the hukou system have promoted large-scale internal migration [83] (*Supplementary Table* 1). Between 2010 and 2020, the number of internal migrants more than doubled (154 million to 376 million). Internal migration, defined according to residence outside of region of household registration [10], is reflected in the decline of the rural population since its 1990 peak, although still >500 million in 2020 (Fig. 4) [84]. China’s agricultural labour force declined rapidly from 1990 to 2019 (from 59·7% to 25·3%) [85].

This flow of rural populations to urban areas has resulted in significant disparities in regional population age structures and proportion of older people. Origin regions have become depleted of younger people due to migration of the young workforce. At the same time there has been return-migration of the older workforce [86]. The main flow of migration is from western and central China to the East [87].

It would be over-simplifying to describe internal migration as purely rural-to-urban. Increasingly, there is counterflow migration, when young adults choose to return to their family in rural provinces from the city [88]. Youths cite a better quality of life in the countryside: cities are associated with ‘city disease’ (*chengshibing*) characterised by high rents, poor air quality and long working days [88]. Expanding internet access, e-commerce and rail links have also helped to increase access to cities, raising income in rural areas. The COVID-19 pandemic crystalised this pattern. In 2020, the total number of migrant workers was 286 million, a decline of five million from 2019 [89].

## Relationship

### Changing family and household structures

The importance of family in China is signalled by the positioning of the surname first, before the given name. Filial piety is at the centre of traditional Chinese culture, where a child is expected to care for their ageing parents [90]. A focus on family-based care for older people remains China’s national policy principle under a 90-7-3 framework, dividing older adults’ care into three groups: 90% are expected to be cared for at home, 7% with support from community-based services, and 3% in an institution, i.e. a care home or nursing home, where needs exceed family and community caregiving capacity [1].

Prior to economic reforms, the traditional agricultural economy and patriarchal-led society provided conditions for filial piety, where children were dependent on their elders for social connections and employment [91]. China’s welfare housing system gave older employees priority for better and bigger housing and allowed their married children to live with them. A replacement policy also gave retiring adults the opportunity to secure employment for their children in the same workplace [91].

**Fig. 5:**
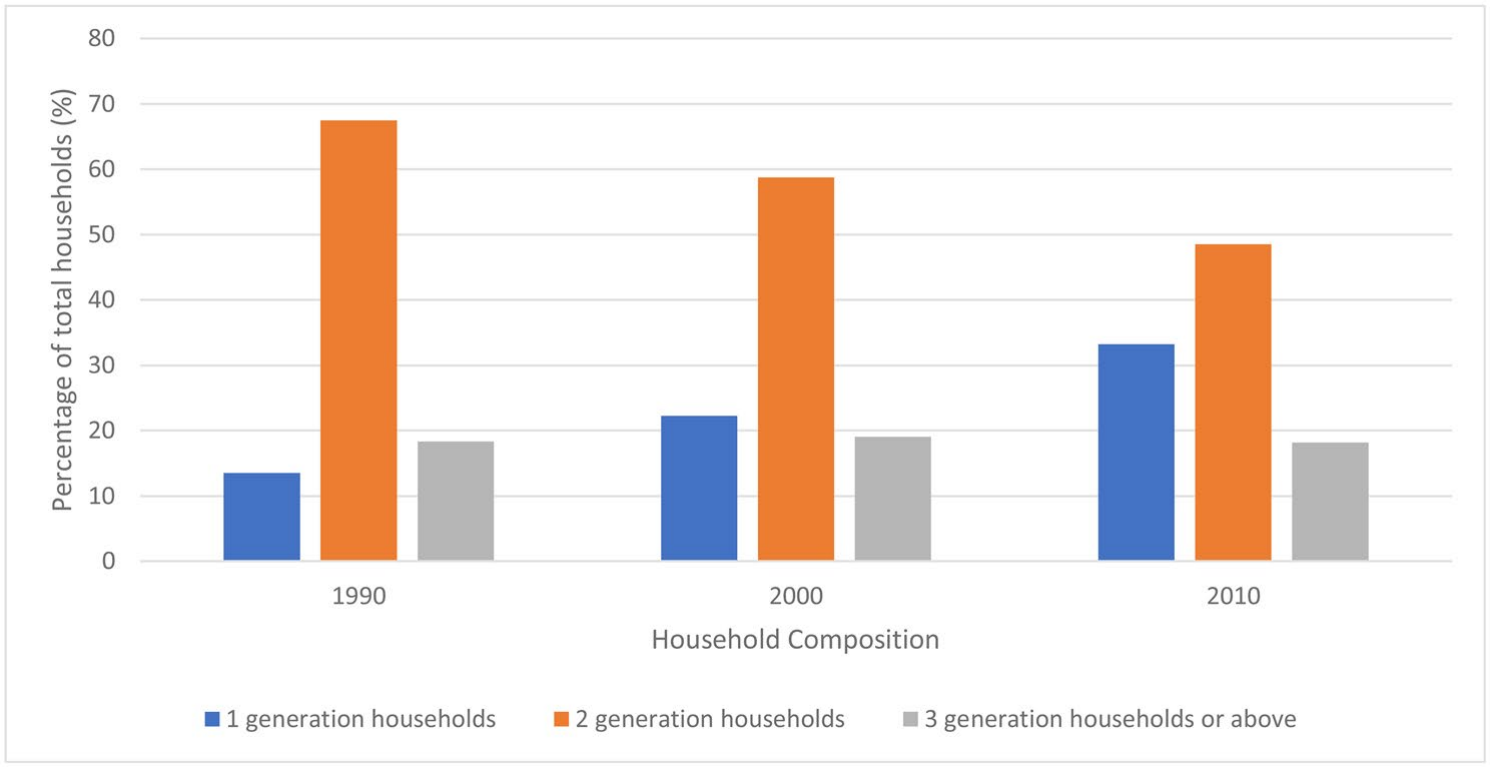
Household composition in China, 1990–2010, percentage of total *Source: Hu et al. Chin J Sociol. 2015.* [93] Footnote: Household composition is defined as individuals living together who may or may not be related by blood or marriage. A one-generation household is defined as a single-person household or only-couple household. A two-generation household is a household of parents and unmarried children, a single parent and unmarried children, a separated parent and unmarried children, parents and married children, or grandparents and grandchildren. A three-generation household constitutes elderly, children and grandchildren.

However, rising incomes and labour mobility have resulted in multi-generational houses becoming rarer and families more dispersed. By 1990, the extended, three-generation household had become unusual, making up less than 20% of households (Fig. 5) [92]. Average household size in 1982 was 4·4 persons, and this declined to 3·1 in 2010 and 2·6 in 2020 [90]. In 2011-12, only one third of those aged ≥60, who did not live with an adult child, had an adult child living in the same neighbourhood [93].

However, migration and hukou registration make it difficult to interpret the census data. Many individuals are not registered at their current address. ‘Residence-registration inconsistency’ rose by 88.5% between 2010 and 2021 (492.8 million currently live somewhere other than their household registration) [57].

## Individual

### Health behaviours

The association between unhealthy lifestyle behaviours and NCDs is well known [94]. China’s Health-related Quality of Life Survey for Older Adults in 2018 reported a high incidence of health risk behaviours in participants aged ≥60, particularly physical inactivity (63%) and unhealthy diet (46%) [95, 96]. Almost half of Chinese adults (47%) do not meet the World Health Organization’s recommendations for fruit and vegetable consumption, with the highest prevalence amongst those ≥65 years old (57%) [96]. There was a two- and five-fold increase in obesity in men and women respectively between 1993 and 2015 [97].

While prevalence of smoking, unhealthy diet, unhealthy weight and physical inactivity is highest among those of lower socioeconomic status, alcohol consumption is associated with higher status [95]. Drinking remains a crucial aspect of social bonding and business interactions for men. Men exhibit higher rates of binge drinking and more days drinking per week than women [98]. 31% of female non-drinkers reported that cultural restrictions were the main reason for abstinence from alcohol [99]. Gendered risk behaviours also extend to smoking: male smoking prevalence in 2015 was among the highest in the world, at 52·9%, compared to 2·7% for women [100].

### Ageing social care policy

These determinants, in combination with demographic changes, are shaping social care policy for the older Chinese population. Relatively few children are available to shoulder the burden of care for the rising number of longer-lived parents and grandparents. According to the China Health and Retirement Longitudinal Study (CHARLS), an individual aged 80–84 has an average of four living children, but those aged 60–64 only have 2·8 [60]. This has led to the emergence of the ‘4:2:1 effect’: couples who are responsible for care of one child and four older parents [74]. Unintended consequences include neglect of elders [101].

To complement traditional family care, residential provision and old age insurance programmes have been scaled up [1]. However, financing of residential care is means tested, with exceptions for some individuals ≥80 years old, with complex needs, or living alone [102]. In most cases, older people pay for formal LTC from savings which depend on children for financial support [1]. Affordability is a challenge for many; the rural population is particularly disadvantaged, lacking pension rights [74].

In response, revision of the *Protection of the Rights and Interests of Elderly People Law* in 2013 (originally passed in 1996) mandates adult children to provide support to their ageing parents, constituting regular visits and attention to their spiritual needs [101]. In 2016, Shanghai’s administration announced that children whose care for their parents is considered insufficient may be put on a credit blacklist, denied a bank account, starting a business, or buying a house [101]. Effectiveness of these measures is unknown. Evaluation relies on older adults to report their children. Fulfilling a quota of visits may be insufficient to meet the needs of all older parents.

### China’s emerging long-term care system

In the face of China’s increasing need for care of older people, the 13th Five Year Plan (2016–2020) made healthy ageing a priority on the national policy agenda. In line with the Sustainable Development Goals, Healthy China 2030 was implemented in 2016 as a blueprint for promotion of equitable health [103]. A Peking University-Lancet Commission details policy initiatives to promote the health and care of older people [104].

At the same time, LTCI pilots, with varying structures of funding and service delivery, were established in 15 cities in 2016 with the aim of providing affordable and accessible care to all. An additional 34 cities were added to the pilot scheme in 2020. A comprehensive analysis by Feng et al. in 2020 identified key strengths and limitations of the LTC system: poor system integration, lack of national assessment and eligibility criteria, varying regulation and care quality, increasing private sector growth, and slow development of home and community-based services (HCBS). The authors also identified vast inequalities in LTC needs and service provision between urban and rural areas [1].

Building on these findings, we undertook a systematic review of evidence since the analysis by Feng et al., and introduction of the second pilot phase, to answer the question: ‘what are the key policy challenges to China achieving an equitable nationwide LTC system for older people?’.

## Methods

Following PRISMA guidelines [105], two reviewers independently searched academic databases: PubMed, EconLit, MEDLINE, Social Science Research Network, Wiley Online Library, Google Scholar, Embase, APA PsychInfo and the China Knowledge Resource Integrated Database (CNKI), using search terms “China” and “long-term care” or “geriatric care” or “elderly care” or “integrated care” and “long-term care insurance” or “long-term care system”.

**Fig. 6:**
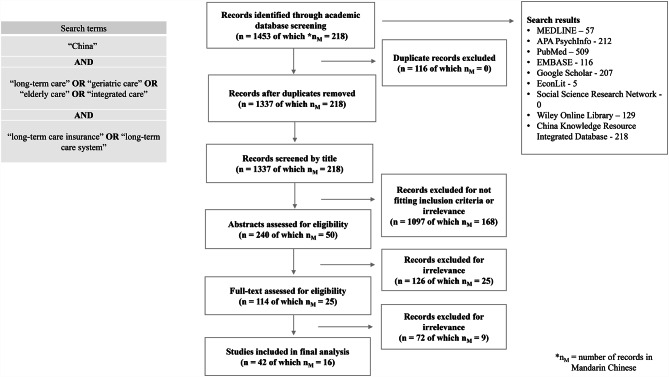
Systematic review flow diagram and search terms

Studies were eligible for inclusion if published in English or Mandarin Chinese between 1st June 2020 and 1st June 2022. Any study design was eligible for inclusion, so as to include policy literature not published in academic journals. One reviewer searched and read studies in English, and the other in Mandarin Chinese. To minimise selection bias, the reviewers and two additional authors read abstracts and agreed the set of included studies. Fig. 6 summarises the search strategy and yield.

When looking at the findings of each study, we considered the possibility of information and selection bias, and quality of the findings, in the context of the overall evidence. Data were extracted by two researchers, independently, to document details of the study characteristics, novel findings, implications and recommendations. Results were validated by two additional authors (*see Supplementary Table 2 for summary of systematic literature review search strategy and selection, according to PRISMA guidelines).*

## Results

42 studies (n = 16 in Mandarin) were included in our systematic review. Table 3 summarises the study characteristics and findings. Four themes emerged: poor quantity and quality of service provision, preferences for family care, inequitable distribution of cost burden, and varying eligibility for LTCI.

**Table 3:** Systematic review findings for Chinese Long-Term Care of older people.

Study	Study design, scope and target population	Novel Findings	Implications and Recommendations
Bao, J., et al. (2022). BioScience Trends. [106]	Review of smart old age care in China.	• “Internet + model” integrates online and offline old age HCBS resources. • Challenges: personal privacy at risk due to use of “big data”, older adults less adept at using digital technology.	• Develop applications that are suited to older adults’ usage. • Enhance management and safety of big data.
Chang, C., et al. (2021). Chinese Social Security Review. [107]	Analysis of policy texts on LTC for older people with disabilities and dementia, published by the State Council from 2011 to 2019.	• National policy documents confuse LTC and concept of filial piety. • Fragmentation of LTC management horizontally between government departments and vertically from national to local level. • Unmatched LTC supply vs. demand.	• Clarify LTC policy aims and responsibilities for the state, market, family and individuals. • Improve governance. • Policies to harmonise supply vs. demand.
Chen, H. & Ning, J. (2022). Health Policy & Planning. [108]	Quasi-experimental study of LTCI on health utilisation and out-of-pocket health expenditure, using data from CHARLS 2011, 2013, 2015, 2018.	• LTCI significantly reduced inpatient out-of-pocket cost by >500 yuan but not outpatient costs. • No. of outpatient visits, inpatient stays and hospitalisations significantly reduced.	• LTCI coverage should be expanded to reduce out of pocket costs.
Chen Y., et al. (2022). Health Economics Research. [109]	Review of 29 LTCI pilot cities.	• Narrow coverage of LTCI. • Multi-channel funding and poor financial sustainability. • Third-party assessments encouraged but often biased. • High integration costs because of fragmentation.	• Principles for development of high-quality LTCI: equality and adequacy. • Collaborate all responsible parties. • Improve LTC service supply capacity.
Du, P., et al. (2021). Research on Aging. [110]	Literature review of government reports, academic databases and reports from international organisations from 2000 to 2019.	• Poor connectivity between regions for service eligibility, funding sources, insurance, management, benefits, subsidies, care costs. • Rural-urban disparities in access. • LTC needs assessment excludes mild and moderate CI and FI. • Preferences for care are family-based. • LTCI coverage is narrow: most areas only cover medical care. • Underutilisation of HCBS (estimated at <10%).	• Increase integration between urban and rural areas. • ‘Person-centred’ care: ensure policies meet LTC needs of all populations. • Consider role of family combined with institutional care. • Extend LTCI coverage. • Use public-private partnerships to link HCBS resources.
Du T., et al. (2022). Health Economics Research. [111]	Analysis of policy texts of 29 LTCI pilot cities.	• Main sources of LTCI funding: individuals, employers, basic medical insurance. • A trend towards employer funding instead of basic medical insurance among urban workers. • Relying on health insurance is not conducive to a sustainable funding system. • Setting funding standards to local economic development is not conducive to formation of a national funding framework. • LTCI predominantly funded on a flat-rate basis; mechanism for dynamic adjustment of rates is missing.	• Funding responsibilities should be reasonably divided. • Move towards proportional funding and establishment of funding criteria to guarantee basic benefits. • Establish a dynamic rate adjustment mechanism.
Fang, E.F., et al. (2020). Ageing Research Reviews. [112]	Review of ageing in China, including long-term care policy.	• Needs assessments tend to be one-off and disconnected to care plans making it hard to allocate appropriate resources. • Investment predominantly in nursing homes resulting in an oversupply of care beds and lack of HCBS. 45% of nursing home beds unoccupied. • Most LTCI schemes are based on social health insurance with different eligibility criteria and benefits packages.	• Care needs should be subject to regular reassessment. • Assessment should include multi-dimensional health status. • Education and training of integrated care managers to coordinate services across public and private sectors. • Link personal health records, assessments of older peoples’ care needs and care costs to integrate data. • Direct more attention to disabled elderly, low socioeconomic status, no family support.
Feng, Z & Glinksaya, E. (2021). China: An International Journal. [113]		• Service quality depends on ability to pay. • Only ≥80 year old age groups are eligible for services in some pilots. • Government invests more in LTC facility construction, beds and subsidising operational costs than cash allowances and consumer service vouchers. • Common feature of all pilots is building on medical insurance programmes. • Ningbo and Guangzhou raise funds solely from UEBMI pooled funds, don’t add individual or employer contributions. • In Changchun, cancer patients can also be eligible for LTCI benefits. Mental illness is not routinely included. • Shanghai is only pilot that specifically sets a minimum age for receiving LTCI benefits, at 60 years old. • Shanghai offers option of either cash or service benefits.	• LTCI fundraising standards should be determined by local need and conditions. • LTCI should not pay for services that are already covered under other existing social insurance systems and avoid duplicate coverage of benefits for the insured. • Government should partner with private-sector enterprises that are qualified to perform disability and needs assessments.
Gruat, J.V. & Chuan, S. (2021). International Social Security Review. [114]	Review of pilot schemes in Qingdao, Changchun, Nantong, Shangrao, Jingmen, Shanghai.	• ~90 million participants, 430,000 service providers, 77% coverage across all pilot schemes. • Health insurance is main funding source, excluding poorer groups who lack cover. • Regional differences in funding, eligibility, services, trained staff, infrastructure. • Beneficiary satisfaction is 82% in western regions vs 69% in eastern regions.	• Dependency insurance should be autonomous social insurance, not part of health insurance. • Costs incurred by beneficiaries should be covered at 70%, to increase access and reduce inequality. • Harmonise regional resource allocation and service delivery.
Han, y. & Shen, T. (2022). International Journal of Environmental Research and Public Health. [115]	Semi-structured interviews with 10 beneficiaries and providers of LTCI in nursing homes and 2 operators at the Medical Insurance Bureau in 4 pilots, Qiqihar, Changchun, Tonghua and Panjin, in North-eastern China.	• Subsidies and policy support are vague for care providers. • Service providers vary across pilots. • 70 years olds unwilling to accept HCBS. • State abolished nursing caregiver qualifications in 2021. • No centralised management of HCBS, difficult to recruit staff to in-home services, more attention paid to institutional care. • When disability levels change, nursing levels cannot be dynamically adjusted and so wastes resources. • LTCI coverage is restricted by medical insurance. • Greater public health expenses due to COVID-19 has brought pressure on medical insurance funds and affected fundraising of LTCI.	• Provide cash subsidies or welfare payments and regular professional training to family caregivers. • Form a nationwide, interconnected information database for services. • Dynamic health monitoring to explore LTC needs of disabled and dementia groups to match supply and demand. • Government should increase financial subsidies for less developed regions. • Incorporate LTCI policies with overall economic and social policies.
He, Y.H., et al. (2021). Chinese Journal of Social Medicine. [116]	Policy analysis of 15 pilot cities: Guangzhou, Ningbo, Chongqing, Anqing, Chengde, Shangrao, Qiqihar, Chengdu, Shanghai, Qingdao, Suzhou, Nantong, Jingmen, Shihezi, Changchun.	• LTCI has a low reimbursement rate, most between 70–80%, which affects the appeal of LTCI.	• Develop LTCI through diversified financing. • Increase LTCI benefits to compensate for low reimbursement, in the form of subsidised services.
Hu, H.W., et al. (2021). Social Security Studies. [117]	Systematic review of the financing framework for care of rural-disabled elderly in China.	• Declining role of family in financing for rural-disability care. • Network of responsibility for financing: government finances basic care services; village collective or mutual aid organisation is pension fundraiser; society and market play a supplementary role.	• Strengthen network and clarify responsibilities for financing: harmonise, training and resource allocation.
Huang, Y.X., et al. (2021). Journal of Nursing Science. [118]	Review of home care services under LTCI systems in China, Japan, Germany and the USA.	• Demand for social LTCI is greater than commercial LTCI in China. • Demand for LTCI in western China is greater than in eastern and central China. • Lack of community elderly care services in western China and elderly access to LTCI.	• Reform existing social insurance system e.g. set up independent LTCI fund. • Improve community elderly care services in western China. • Increase public awareness of LTCI.
Jing, G., et al. (2021). Journal of Risk Analysis and Crisis Response. [119]	Literature review of Shanghai LTCI pilot. Forecasting model using data from individuals ≥60 years old in 2004–2017.	• Participants in LTCI pilot: 234,000 (2018) and 493,000 (2019). • Population ≥60 years old receiving nursing services: 5% (2018) and 10% (2019). • Shortage of nurses, low salaries, few nursing institutions, exacerbated by COVID-19.	• High unmet need. • Increase the number of people receiving care. Increase nursing salaries and training.
Li X. (2021) Shandong Social Sciences. [120]	Policy Simulation Analysis using data from CHARLS, 2011–2015.	• LTCI with an hourly subsidy and urban-rural coordination mechanism provides more stable risk protection than a flat-rate subsidy. • Strong demand for LTCI.	• Integrate an urban-rural financing approach. • Funding from multiple insurance mechanisms is more in line with characteristics of China's elderly.
Liu, H., et al. (2021). European Journal of Ageing. [121]	Cross-sectional study conducted in August 2017. Interviews with 6997 adults aged ≥ 60 years-old in Shandong province.	• Age, education, socioeconomic status, regional distribution, ADLs, loneliness had significant associations with preferences for LTC: family-based care (89%), institutional care (8%) and HCBS (3%). • Most participants knew nothing about HCBS.	• Consider preferences for LTC. • Improve quality of family care. • Increase older adults’ awareness of HCBS.
Liu, H. & Hu, T. (2022). Archives of Public Health. [122]	Difference-in-differences (DID) method for LTCI policy using survey data from CHARLS 2013, 2015 and 2018.	• Number of hospitalization days significantly reduced; self-rated health improved among older adults. • Monthly outpatient reimbursement expenses and annual inpatient reimbursement expenses increased by >4000 yuan/year for older adults. • Most pilots only protect severely disabled who have a higher coverage in overall funding. • LTC services ignore needs of rural disabled and do not have service capacity in rural areas.	• Address needs of moderately disabled individuals. • Improve supply of LTC services in rural areas.
Liu Z., et al. (2022). Medicine and Society. [123]	Literature review of 29 LTCI pilot cities.	• Inconsistent eligibility criteria. • Assessment tools are dominated by single-type indicators and lack comprehensive assessments. • Shortage of assessment agencies and assessors, process lacks effective management.	• Develop a comprehensive LTCI assessment tool. • Improve professional standards of assessment agencies and caregivers. • Strengthen supervision and management of assessment process.
Lu, B., et al. (2020). China Economic Review. [124]	Cost evaluation of Qingdao LTCI pilot using data from recipients who entered the programme in 2015.	• Successfully integrated LTC model. • Reduced social hospitalisation: probability of using in-patient services declined by 12%. • Increased LTC service spending offset by decreased inpatient services spending; overall decline by >10,000 RMB (1500 US$, 1200 GBP). • Eligibility dependent on ADL score <60 and a diagnosed medical condition.	• LTC optimises resource allocation and alleviates hospital overcrowding. • LTCI provides cost-efficient care for the disabled by reducing out of pocket expenses. • Expand eligibility for mild and moderate conditions to increase access.
Luo J., et al. (2022) Medicine and Society. [125]	Survey of senior citizens in Shanghai covered by LTCI.	• Overall LTCI satisfaction higher in Shanghai than other pilots. • The higher the medical expenses, the lower the satisfaction with LTCI. • Older people would like longer service hours.	• Provide more medical care for older people with higher medical expenses. • Reasonable extension of the length of care.
Peng, R. et al. (2022). International Journal of Environmental Research and Public Health. [126]	Review and coupling coordination model of LTCI policy documents issued by the General Office of the Chinese People’s Government and Human Resources and Social Security Bureaus of pilot cities.	• All pilots cover UEBMI; some expanded to URRBMI. • Most cover institutional and home care – Changchun and Ningbo only cover institutional care. • Shanghai, Qingdao, Nantong have highest policy strength and coordination, in line with local economic development and population structure.	• Broad coverage of LTCI should be adopted to improve equity and accessibility of care. • Other cities should study Shanghai’s LTC policy. • Local government should determine the level of LTCI funding based on local economic development.
Peng, R., & Wu, B. (2021). Research on Aging. [127]	System dynamics simulation and policy scenario modelling for adults aged ≥60 with at least one ADL, from 2015 to 2035.	• Low capacity of community-based care for disabled older people, especially in rural areas. • Increasing LTCI compensation and capacity of institutional and community-based care would decrease % of disabled old adults cared for by family members from 93% (2015) to 64% (2035).	• Adjust resource allocation between institutions and community. • Policies should balance family caregiving burden and LTC expenditures.
Shu Z., et al. (2022). Population and Development. [128]	Cohort study using CLHLS 2014 and 2018 data.	• LTCI can "squeeze out” family financial support. • LTCI plays a positive role in intergenerational relationships.	• Provide family caregiver support policies. • Promote synergies between LTCI governance and social protection systems.
Sun, Y.X., et al. (2021). Chinese Nursing Research. [129]	Review of LTC models in the USA, Japan and China.	• No unified standard for disability assessment and grading; ADL scale mostly used. • Different needs among old age groups are not paid attention to.	• Multi-dimensional disability assessment system should be built.
Tang W., et al. (2021). Journal of Finance and Economics. [130]	Cohort study using four waves of CLHLS data from 2008 to 2018.	• LTCI contribution rate from employees higher than that of residents.	• Form a LTCI pay-as-you-go system. • Make residential care at low cost, which is under the greatest funding pressure.
Tang, Y., et al. (2022). Frontiers in Public Health. [131]	DID method evaluating LTCI using data from CHARLS, 2011, 2015 and 2018.	• LTCI reduced number of outpatients and inpatients by 0.2 and 0.1 per year. • LTCI cut outpatient and inpatient expenses by 24% and 20% per year. • LTCI improved self-rated health and ADLs.	• Integrate grading diagnosis and treatment with LTCI to match medical and nursing systems. • Improve training of care service teams.
Wang, B. & Xu, L. (2022). Journal of Healthcare Engineering. [132]	Review of “Internet Plus” community smart care service platform.	• Uses big data and smart mobile devices to monitor older people in real time. • Allows community centres to provide timely and accurate information for older adults’ service needs. • Low willingness of older adults to accept, privacy leakage issues. • Industry service standards have not been developed. • Connectivity and integration of resources is weak due to service fragmentation.	• Government policies should integrate smart elderly services and collaborate service providers. • Improve elderly technological literacy. • Increase efforts to promote positive role of technology. • Develop easy-to-operate platforms. • Reward and punishment mechanisms to incentivise providers to prioritise care quality.
Wang, K., et al. (2021). International Journal of Health Planning and Management. [133]	Content analysis of 12 major Chinese news portals in 2018.	• Most frequently identified LTC issue: few qualified professionals (47%). • Few service types, low quality services, poorly integrated care, unstable LTC economic model e.g. for private investors, poor public understanding, organisational fragmentation.	• Private investors should evaluate their ability to recruit and train care staff, integrate care and expand profit patterns in HCBS. • Government should formulate policies for private investors and promote public awareness of HCBS.
Wang, Q., et al. (2021). Social Science and Medicine. [134]	Discrete choice experiment with 1067 community residents in Shenyang and Dalian, Liaoning province.	• Strong preferences for LTCI. • Factors driving preferences: coverage ceiling, HBCS reimbursement, individual premiums. • Poor coverage of complex daily assistance packages (home environment adaptation, dementia care).	• Consider how to increase attractiveness and sustainability of LTCI.
Wang, C., et al. (2022). Frontiers in Psychology. [135]	3513 questionnaires from older Chinese adults in 7 LTCI pilot cities.	• Older adults living with children are 20% less likely to choose nursing homes than those living alone. • Male older adults are 30% less likely to choose nursing homes. • Older adults with more hospitalisations more likely to choose nursing homecare. • Those with greater monthly income, higher education level or a nursing home nearby are more willing to choose nursing home care. • Insured older adults are 1.5x more likely to choose nursing home care.	• Expand LTCI coverage. • Integrate interdisciplinary professionals in nursing homes to provide high-quality services. • Promote medical services in nursing homes. • Locate nursing homes in communities. • Improve design of nursing homes to create sense of homeliness.
Wei, Y., & Zhang, L., (2020). International Journal of Environmental Research and Public Health. [136]	Questionnaire surveys with 3260 elderly people aged ≥60 in six districts of Xiamen province.	• 82% chose home-based care, 13% chose institutional care with integrated nursing and medical services (up from 3% in 2013), 5% chose community-based care. • Older age, higher education level, living in rural areas, better economic status, those cared for by others (other than spouses) are more willing to accept integrated services.	• Consider needs of different demographics. • Strengthen family care and integrated care policies. • Improve awareness of integrated care. • Encourage implementation of integrated care in rural areas.
Wu, B., et al. (2021). Research on Aging. [137]	Literature review of 6 recently peer-reviewed articles (2020) addressing issues related to LTC in China.	• COVID-19: reduced quality of community-based care, patients delayed moving into institutions, increased operational costs, stretched funding for LTC, high staff turnover rates. • Social isolation common for disabled older adults but many lack knowledge of and access to mobile technology.	• Regulate community-based care. • Improve LTCI benefits for disabled older people. • Increase wages to retain and attract staff. • Improve access to mobile technology. • Develop person-centred applications with input from older adults.
Yang, W., et al. (2021). Research on Aging. [138]	Qingdao pilot: analysis of 47 qualitative interviews conducted in 2016 with government officials, care providers and family members of service users.	• Eligibility excludes people with mild and moderate cognitive impairment. • Poor public awareness of eligibility and service entitlement leads to unequal and unfair treatment. • LTCI funds mostly from social health insurance means there are different benefits for those with same needs. • Disparities in financial burden: poorer service users likely to incur high co-payments.	• Widen eligibility to include those with moderate cognitive impairment. • Funding needs of low socioeconomic groups. • Improve accessibility of information on entitlement and eligibility. • Consider mandatory premium contributions.
Yang Y., et al. (2022). Chinese Journal of Health Policy. [139]	Policy analysis.	• China's LTC policy does not align with the service system.	• Establish independent LTCI financing. • Consolidate and use resources already available to fund LTC.
Zhang, Q., et al. (2020). BMC Geriatrics. [140]	Review of China’s policies on smart home elderly care.	• Smart care is policy-driven, not-demand driven. • Older adults have little interest or understanding. • Most older people regard smart care as a welfare product whereas providers want to make a profit. • No industry standards or national regulation. • Multiple government departments are jointly responsible for supervision.	• Explore older adults’ willingness to use smart care • Form technical standards. • Combine existing public and private smart home platforms to optimise resource allocation and management. • Encourage development of new technologies to reduce cost of products and make smart care accessible and acceptable for older people.
Zhang, L. (2021). Frontiers in Public Health. [141]	System dynamics model of LTCI financing system using data from Xiamen Special Economic Zone Yearbook and field study.	• Without any intervention, revenue and expenditure of LTCI funds from 2020 to 2030 will increase year on year by 3.7 times and 8.8 times, respectively. • After 2029, expenditure > revenue amounting in an LTCI deficit. • Highlights urgency of improving LTCI financing system and establishing a unified LTCI financing mechanism.	• Increasing the individual payment rate can delay deficit. • Increasing government financial subsidies and enterprise contribution rates can prevent deficit. • Implement a paying policy for urban retired employees which can increase revenue of LTCI funds and maintain its stability and improve fairness. • Share funding responsibilities between individuals, enterprises, government.
Zhang, Z.Y., et al. (2021). Chinese Health Service Management. [142]	Policy analysis: integration of medical and care services between the 13th Five-Year Plan (2016-20) and the 14th Five-Year Plan (2021-25).	• Needs assessments exclude many requiring care and don’t consider financial care burden. • Management of integrated care fragmented and inefficient.	• Form a hierarchical assessment of needs and link this to charging standards and service supply of institutions and HCBS. • Establish a big data platform for health management of older people.
Zhang, Q., et al. (2020). Healthcare. [143]	Cross-sectional analysis of CLHLS, 2018. Sample of 1617 disabled adults aged ≥60 with children or children-in-law as primary caregivers.	• Rural residence and lower socioeconomic status groups associated with under met care needs. • Family caregiving is highly valued. • COVID-19 affected family care model: no support measures introduced for isolated people in family care, poor access to medicines.	• Promote financial assistance to the oldest old, particularly in rural areas, to enhance access to services. • Policies to support family caregivers: provide care skills training, respite services, psychological counselling, pilot an allowance.
Zhang, J., et al. (2022). Psychogeriatrics. [144]	Cross-sectional survey of 1011 elderly residents ≥60 years old living at home with disabilities in Kunshan, Suzhou province, 2018.	• 80% chose living at home as their most preferred living arrangement. • Individual income was a significant predictor of preferred living arrangement. • Those with a monthly income of <3000RMB were less likely to choose living in a nursing home over at home. • Older adults with <2 children were more likely to choose living in a nursing home or healthcare institution than at home as they were likely to have better financial support.	• Give special attention to older people with low individual income. • Promote use of home-based services to suit preferences. • Limitation: study excluded those with severe cognitive impairment.
Zhao, R., et al. (2021). Journal of Health Care Organization, Provision and Financing. [145]	Cross-sectional study. Questionnaires with residents aged ≥65 in Chongqing.	• 85% choose home-based care: family care (56%) family and community care (29%). • Preferences attributed to monthly income, number of children, insurance, health status, distance to children.	• Consider preferences for care, with reference to 90-7-3 policy guidelines • Older adults are a heterogeneous group. • Encourage doctors and nurses to work in institutions to provide integrated services.
Zheng X., et al. (2022). Medicine and Society. [146]	Policy analysis of LTCI pilots.	• Restricted scope of coverage. • Lack of unified assessment criteria. • Funding mechanisms being explored by pilot cities.	• Attention should be paid to protecting people with different levels of disability and dementia. • Content and types of services should be expanded e.g development of psychiatric support services.
Zhou, W., et al. (2021). Health Economics Research. [147]	Systematic review of LTC policies for older people in China.	• Poor resource integration due to multi-leadership and management fragmentation. • Service system does not adequately meet LTC needs.	• Related departments to jointly set up a working group. • Identify target populations for LTC and develop service capacity.

## Key findings

### Service quality and provision

Service providers recognise the limited scope of LTC services, and low quality, among major difficulties to be addressed as the LTC market expands [133].

The time-intensive nature of LTC requires a large, skilled workforce [107]. However, care workers are given low social status, with limited policy support, attracting few to the profession [133, 115]. High staff turnover rates since the emergence of COVID-19 have further diminished care quality [119, 137]. Increasing salaries and training opportunities, to retain and attract staff, have been proposed to meet the increasing demand for care [119, 137].

Distribution of LTC resources are skewed towards urban areas, despite an urgent need for services in rural regions, where older people have poorer health and are more likely to live alone [136, 122]. Despite the 90-7-3 LTC policy goal, investment is predominantly in institutional care, neglecting HCBS. Institutional care beds are oversupplied and met with low occupancy rates [112]. Current uptake of HCBS is low (<10% in some areas) [110]. In many pilots, HCBS was the least popular mode of delivery amongst older adults, and had limited capacity to care for those with disabilities [136, 110, 145]. Management fragmentation and multi-leadership of LTC at national and local level contribute to this problem [107, 147]. There is no centralised management of HCBS [115].

Encouraging nurses to work in both HCBS and institutional settings, and training “integrated care managers” to coordinate care resources, could improve quality and encourage greater use [112, 145, 148]. Government awareness programmes of HCBS could also enhance uptake [118, 121, 149]. Improved governance, through joint-working across regional government departments, could help to understand where resources are most needed [107, 147, 108].

### Preferences for care

Within regions, heterogeneity of income, wealth and living conditions mean that LTC systems face expectations arising from a broad range of care needs and preferences for mode of delivery [136, 145]. However, differing needs of older adult groups are not being paid attention to [129]. A ‘person centred care’ approach is required [137, 110].

Preferences shift towards formal care in more economically developed regions and large cities [110]. In Xiamen, those with a higher education level and better economic status were more willing to accept integrated medical and care services [136]. In Shanghai, the number of people receiving formal care increased over two-fold between 2018 to 2019 [119].

Despite these regional differences, preferences for family care remain widespread across China and are largely dependent on participants’ distance to and number of children [145, 150]. In most cases, those with a greater number of children prefer home-based care [150, 135, 144]. In Suzhou, those with more children were able to afford institutional care [144]. Improving the design and proximity of nursing homes to communities could facilitate the willingness of older people to enter [135].

In the COVID-19 pandemic, where institutions were forced to postpone entry of new patients, family care was essential [137, 143]. However, many older people who relied on family care were isolated at home, unable to use mobile technology [137]. Poor technological literacy, service standards, privacy fears and unwillingness to pay for smart old age care services contribute to low uptake. Improving older adults’ technological literacy and implementing incentives for providers to deliver affordable, quality care, could facilitate the safe use of smart services and suit preferences to age at home [151, 106, 132, 152]. Strengthening family care policies through regular skills training, piloting a family care allowance, welfare payments and cash subsidies, are also important policy proposals [143, 128, 115].

### Eligibility

Eligibility criteria for LTCI vary considerably across pilots, often excluding those with mild and moderate cognitive impairment [112, 110]. Potential service users are often unclear about their service entitlement meaning older adults with the same level of disability are unable to access the same services [110, 138]. Assessment tools are dominated by a single-type indicator [123].

Adequate information on eligibility should be made available to the public [138]. A unified standard of disability assessment is required, so that the extent to which mild and moderate impairments are insured can be understood by policymakers and the public [129, 112]. Regular reassessment is needed to develop personalised care plans and allocate adequate resources [112, 115]. A big data platform, recording multi-dimensional disability assessments, could facilitate nationwide standardisation [129].

### Financing

An unstable economic model is a key challenge for China’s LTC system [133, 143]. Undoubtably, the search for a nationwide financing model is helped by the diverse range of LTCI pilots [112, 146].

Qingdao’s pilot reduced ‘social hospitalisation’ by 12% when patients who did not require hospital inpatient services were transferred to institutional or home-based care. Increased LTC service spending was offset by decreased inpatient service spending, with a net cost reduction of >10,000 RMB (1,500 US$, 120 GBP) per person [124]. LTCI significantly reduced utilisation of outpatient and inpatient services according to CHARLS, cutting expenses by 24% and 20% per year, respectively [108, 122, 131]. However, LTCI expenditure is expected to exceed revenue after 2029 in Xiamen [141].

Cost-efficiency of pilots’ varies due to their reliance on social health insurance (SHI) as a main source of funding, which despite near universal coverage, is based on regional socioeconomic characteristics [115, 110, 138, 126] All pilots cover participants in the Urban Employee Basic Medical Insurance (UEBMI); some have extended cover to urban and rural residents (URRBMI) [126]. Reimbursement rates for UEMBI are fixed at 90%, while rates are 70–80% for URRBMI. As such, poorer families face exclusion from formal LTC because they are unable to meet out-of-pocket charges [114, 135].

Nationally, this hazard is being tackled with government-funded support for providers if their services are made available to low-income users. However, fee-for-service arrangements for paying providers is widespread, incentivising unnecessary care and excessive costs. Providers often reject potential service users if they are deemed to incur high costs.

In response, the second pilot phase announced LTCI independence from SHI [118]. However, most cities continue to rely on surplus health insurance [126, 111]. Greater public health expenses since COVID-19 has further increased pressure on SHI funds, impacting LTCI fundraising [115]. Government subsidies, a unified move towards proportional funding and criteria to guarantee basic benefits, particularly for those with low economic status, could be beneficial [111, 143, 120, 115]. Germany and Japan supplement SHI with mandatory contributions from employed people of working age [129]. China may adopt this approach to reduce reliance on SHI. Mandatory contributions from employers and urban retired employees could also increase revenue of LTCI funds, and improve system fairness [141]. However, implementation may prove difficult as employers are already required to make contributions to other social insurance programmes [113].

## Discussion

Dramatic improvements in life expectancy, adult and child mortality have resulted in a seismic shift in China’s population from a predominantly young to a rapidly ageing population within a period of 50 years. By 2050, it is projected that China’s ≥65 year old population will exceed that of the UK and the USA, and approach that of Japan.

Using a socioecological framework, we examine reasons for why China’s population health has rapidly improved from 1970 to 2020. Economic and educational reforms have resulted in significant reductions in poverty and greater labour mobility.

However, longer life expectancies come with an increased burden of age-related conditions. Following the country’s economic development, NCDs have largely replaced communicable diseases, influenced by a high prevalence of smoking, alcohol consumption and obesity. An estimated 33 million older adults (aged ≥60) are living with disability, set to increase further by 2050 [153]. The impact of COVID-19 on disability is still unknown. Emerging evidence shows high rates of cognitive impairment and post-viral symptoms among older adults.

Crucially, China is still a middle-income country. High-income countries such as Japan, the USA and the UK are struggling to meet the care needs of their older populations, and China faces the additional challenge of ‘being old before being rich’. Mass internal migration of the younger population, in the context of changing fertility policies, have dispersed families and shaken traditional models of care. A huge number of older people face the possibility of inadequate care. For a population aged ≥65 which is expected to reach 366 million in 2050, the scale of this issue is immense.

In response, national policy has focused on rapidly scaling up provision of equitable and affordable care for all. Since 2016, China has implemented 49 LTCI pilots across a range of cities to find the most suitable LTC model for its older population with varying care needs. The most recent evaluation of these pilots was conducted by Feng et al. in 2020, who highlighted strengths and weaknesses based on the first 15 pilots. An additional 34 cities were added to the pilot scheme in 2020. We undertook a systematic review of Chinese and English evidence, to include findings from the second pilot phase.

A strength shared across pilots is their reliance on funds from a nearly universal SHI, maximising the pool of risk. However, shallow coverage in terms of services and reimbursement rates means high out-of-pocket charges for users. Benefits packages are biased towards urban employees, where more generous packages are applied to participants of UEMBI over URRBMI. Proportional funding and mandatory employment contributions could assist lower socioeconomic groups who are unable to afford care. In Germany, eligibility for LTC requires almost all workers and pensioners to contribute to social insurance [154]. In Japan, contributions are based on earnings, levied on people aged ≥40 [155]. These compulsory schemes have done well to address unmet need among poorer groups [156].

Narrow eligibility criteria means only a small percentage of the disabled population have access to LTCI. The criteria in most pilot studies exclude mild and moderate cognitive impairments. In contrast, the Japanese system provides support for those ≥65 years old based on need alone, including mild impairments and independent of income and wealth, and LTCI cover includes those aged 40–65 with Parkinson’s disease, ADRD or stroke [156]. Forming a nationwide hierarchical assessment of needs, with links to charging standards, could extend coverage and improve equity of China’s system. Regular and multi-dimensional disability assessment can also ensure allocation of appropriate resources.

Addressing current and future needs requires the sector to become more attractive. Increasing wages for LTC staff in the USA promoted greater recruitment, longer tenure and lower turnover [157]. This approach seems suitable in the Chinese context, where high income is the most important criterion for choosing a job [158]. Investment in LTC training programmes for students and current staff increased the number of LTC workers in Japan by 20% between 2011 and 2015 [157]. The Netherlands has developed coaching programmes for its staff, to promote prevention of burnout, while self-managed teams give nurses more autonomy [157].

Where family care remains the preferred mode of delivery, greater steps can be made to support family caregivers. Caregiver stress in China is associated with financial costs of providing hands-on care [159]. Cash subsidies and welfare payments, like the Carer’s Allowance in England, can support family caregivers [160].

Technology could also be an effective solution to relieving workforce pressures. A systematic review found that remote interventions for patients with rheumatic and musculoskeletal disorders improved patient-reported outcomes including quality of life, activity, and disease severity [161]. Smart care particularly satisfies preferences to age at home [162, 163]. Where older adults are at a high risk to serious illness from COVID-19, digital technologies can also provide alternatives to face-to-face contact. However emerging literature has shown poor digital engagement among LTC residents during COVID-19 [164]. Where technological illiteracy and reluctance is high among older adults, greater incentives and educational tools are required.

Despite a high proportion of COVID-19 outbreaks in LTC facilities, limited literature has explored China’s policies to support LTC. National guidelines announced a one-off allowance to LTC providers, for deploying additional workers and reimbursing overtime, to ensure continuity of services [165]. Older people who are unable to access unpaid carers have been promised home-based services or temporary residential care. Future research could benefit from exploring the effectiveness and degree of implementation of these policies.

### Strengths and limitations

The findings of this review are from a multitude of study designs and provide evidence from different pilots. A wide range of English and Chinese databases were screened to capture as many records as possible. A large number of records was included (n = 42). However, our analysis depends on the accuracy and completeness of information in the published literature. Reports only relate to the situation at the time of writing and therefore do not fully reflect the ongoing pilot experience.

## Conclusions

Population ageing in China is driving an increase in need for social care. More than one quarter of the population will be older by 2050 (≥65 year old population projection 366 million). Rapid economic development and increased access to education has led to widescale internal migration. Geographical fragmentation of families poses considerable challenges to the traditional family care model, at the same time as rapidly increasing age-related disability.

China’s main policy initiative has focused on piloting alternative LTCI systems. This is a rational, quasi-experimental approach to determine how best to meet the care needs of older people in a middle-income country with a substantial proportion of low-income households. China’s pilots can provide useful lessons for other middle-income countries with rapidly ageing populations.

Our systematic review highlights significant challenges in the provision of equitable care which suits the preferences of its users. Inconsistent financing, varying eligibility and reduced service capacity are key challenges. Inequity is a consistent theme across pilots, understandable in the context of considerable geographic and socioeconomic inequalities.

## Electronic supplementary material

Below is the link to the electronic supplementary material.

Supplementary Material 1

## Data Availability

All data generated or analysed during this study are included in this published article [and its supplementary information files].

## Abbreviations

ADLs: activities of daily living
ADRD: Alzheimer’s disease and related dementia
CVD: cardiovascular disease
CHARLS: China Health and Retirement Longitudinal Study
CKNI: China Knowledge Resource Integrated Database
GBD: Global Burden of Disease
GDP: Gross Domestic Product
HCBS: home and community-based services
LTC: long-term care
LTCI: long-term care insurance
NCD: non-communicable disease
NRA: normal retirement age
OADR: old-age dependency ratio
COVID-19: SARS-CoV-2
SHI: social health insurance
TFR: total fertility rate
UK: United Kingdom
USA: United States of America
URRBMI: Urban and Rural Resident Basic Medical Insurance
UEBMI: Urban Employee Basic Medical Insurance
DID: Difference-in-differences
CLHLS: Chinese Longitudinal Healthy Longevity Survey
DALYs: disability adjusted life years
