# Supplementary material for: Growing old in China in socioeconomic and epidemiological context: systematic review of social care policy for older people
Source: BMC Public Health. 2023 Jun 30;23:1272. doi: 10.1186/s12889-023-15583-1 (PMC10311713; doi:10.1186/s12889-023-15583-1)
Supplement: Supplementary file 1 — Supplementary Material 1 [file 12889_2023_15583_MOESM1_ESM.docx]

**Supplement to: Growing old in China in a socioeconomic and epidemiological context: a systematic review of social care policy for older people**

**APPENDIX**

Contents

[Supplementary table 1: Top 10 destination cities for internal migration, 2010. 2](#_Toc118211620)

[Supplementary table 2: Summary of systematic literature review search strategy and selection, according to PRISMA guidelines 3](#_Toc118211621)

# **Supplementary table 1: Top 10 destination cities for internal migration, 2010.**

| Rank | City | Region | Total population, millions | Internal migrant population, millions | Migrants as proportion of total population |
| --- | --- | --- | --- | --- | --- |
|  |  |  |  |  |  |
| 1 | Shanghai | East | 23.0 | 11.0 | 0.50 |
| 2 | Beijing | North | 19.6 | 8.9 | 0.45 |
| 3 | Shenzhen | South | 10.4 | 8.3 | 0.80 |
| 4 | Dongguan | South | 8.2 | 6.4 | 0.78 |
| 5 | Guangzhou | South | 12.7 | 5.4 | 0.43 |
| 6 | Suzhou | East | 10.5 | 4.5 | 0.43 |
| 7 | Chengdu | Southwest | 15.1 | 3.9 | 0.26 |
| 8 | Tianjin | North | 12.9 | 3.9 | 0.30 |
| 9 | Foshan | South | 7.2 | 3.6 | 0.50 |
| 10 | Wenzhou | East | 9.1 | 3.2 | 0.35 |

*Source:* *Lu & Xia. Asian Development Bank Institute. 2016.^84^*

# **Supplementary table 2: Summary of systematic literature review search strategy and selection, according to PRISMA guidelines^106^**

| Section and topic | Systematic review |
| --- | --- |
| Title | Our title reads: “Growing old in China in a socioeconomic and epidemiological context: a systematic review of social care policy for older people”. |
| Abstract | The abstract outlines our methods, eligibility criteria, research question, and main findings of the systematic review. |
| Introduction | We provide an explicit statement on our objectives and rationale of our systematic review in the Background on page 6:  *“Comparison of China with Japan, USA, UK, and India, demonstrates a need to understand the social policy implications of population ageing in both generic and unique aspects of the Chinese context.*  *We aim to achieve this by summarising China’s key demographic and epidemiological trends relevant to ageing and health from 1970 to present, using official and population-level statistics, followed by an exploration of the key determinants of China’s rapidly improving population health within a socioecological framework.^3^ We then carry out a systematic review to answer the question: ‘what are the key policy challenges to China achieving an equitable nationwide long term care (LTC) system for older people?’.”*  This is further expanded on page 19/20:  *“In the face of China’s increasing need for care of older people, the 13^th^ Five Year Plan (2016-2020) made healthy ageing a priority on the national political agenda. With the aim of providing affordable and accessible care to all, LTCI pilots, with varying structures of funding and service delivery, were established in 15 cities in 2016. An additional 34 cities were added to the pilot scheme in 2020.*  *A comprehensive analysis by Feng et al. in 2020 identified key strengths and limitations of the LTC system: poor system integration, lack of national assessment and eligibility criteria, varying regulation and care quality, increasing private sector growth, and slow development of home and community-based services (HCBS). The authors identified vast inequalities in LTC needs and service provision between urban and rural areas.^4^*  *Building on these findings, we undertook a systematic review of evidence since the analysis by Feng et al. and the introduction of the second pilot phase, to answer the question: ‘what are the key policy challenges to China achieving an equitable nationwide LTC system for older people?’.”* |
| Methods | Figure 6 summarises the information sources screened, search strategy, selection criteria and data extraction process. It includes the number of records identified in the search from each database and the number of records included in the final review.  We provide details of our Methods on page 20:  *“Following PRISMA guidelines (Supplementary table 2), two reviewers independently searched academic databases: PubMed, EconLit, MEDLINE, Social Science Research Network, Wiley Online Library, Google Scholar, Embase, APA PsychInfo and the China Knowledge Resource Integrated Database (CNKI), using search terms “China” and long-term care” or “geriatric care” or “elderly care” or “integrated care” and “long-term care insurance” or “long-term care system”.*  *Studies were eligible if published in English or Mandarin Chinese between 1^st^ June 2020 and 1^st^ June 2022. One reviewer searched and read studies in English, and the other in Mandarin Chinese. To minimise selection bias, two additional authors validated studies chosen. The search strategy and yield are summarised in Figure 6.”* |
| Results | Table 3 presents our results, including the study characteristics (study design, scope and target population), findings and recommendations. |
| Discussion | We provide a general interpretation of our results on pages 25-27 and discuss implications of these results for the policy progress of long-term care in China. |
| Other information | Funding and competing interests are outlined in the declaration at the end of the manuscript. |
